# Mortality and severe morbidity of very preterm infants: comparison of two French cohort studies

**DOI:** 10.1186/s12887-019-1700-7

**Published:** 2019-10-17

**Authors:** Anais Godeluck, Patrick Gérardin, Victorine Lenclume, Corinne Mussard, Pierre-Yves Robillard, Sylvain Sampériz, Valérie Benhammou, Patrick Truffert, Pierre-Yves Ancel, Duksha Ramful

**Affiliations:** 1 CHU de la Réunion, Saint Denis, Réunion; 2 INSERM CIC1410 Epidémiologie Clinique, CHU de la Réunion, Saint Pierre, Réunion; 3 CHU de la Réunion, Saint Pierre, Réunion; 4 Centre d’Etudes Périnatales de l’Océan Indien (CEPOI), Université de la Réunion, EA 7388 Saint-Denis, France; 5 INSERM U 1153, CHU Cochin Hôtel Dieu, Paris, France; 6 CHU Lille, EA 2694 Public Health, Epidemiology and Quality of Care unit, F-59000 Lille, France; 7 Université Paris Descartes, Paris, France; 8 URC – CIC1419 Plurithématique, Cochin Hôtel Dieu, Paris, France; 9 Postal address: Neonatal and pediatric intensive care unit, Félix Guyon Hospital, CHU de La Réunion, Allée des Topazes, CS 11021, 97400 Saint-Denis Cedex, La Réunion France

**Keywords:** Preterm, Very low birth weight, Morbidity, Mortality, Bronchopulmonary dysplasia, Intraventricular haemorrhage, Periventricular leukomalacia, Necrotising enterocolitis, Retinopathy of prematurity, Cohort studies

## Abstract

**Background:**

In Reunion Island, a French overseas department, the burden of preterm birth and perinatal mortality exceed those observed in mainland France, despite similar access to standard perinatal care. The purpose of the study was to compare the outcome of two cohorts of NICU-admitted very preterm infants born between 24 and 31 weeks of gestation (WG): the registry-based OGP (Observatoire de la Grande Prématurité, Reunion Island, 2008–2013) cohort, and the nationwide EPIPAGE-2 (mainland France, 2011) observational cohort.

**Methods:**

The primary outcome was adverse neonatal outcomes defined as a composite indicator of in-hospital mortality or any of three following severe morbidities: bronchopulmonary dysplasia (BPD), necrotising enterocolitis, or severe neurological injury (periventricular leukomalacia or grade III-IV intraventricular haemorrhages). Logistic regression modelling adjusting for confounders was performed.

**Results:**

A total of 1272 very preterm infants from the Reunionese OGP cohort and 3669 peers from the mainland EPIPAGE-2 cohort were compared. Adverse neonatal outcomes were more likely observed in the OGP cohort (32.6% versus 26.6%, *p* <  0.001), as result of both increased in-hospital mortality across all gestational age strata and increased BPD among the survivors of the 29–31 WG stratum. After adjusting for gestational age, gender and multiple perinatal factors, the risk of adverse neonatal outcomes was higher in the OGP cohort than in the EPIPAGE-2 cohort across all gestational age strata.

**Conclusions:**

Despite similar guidelines for standard perinatal care, very preterm infants born in Reunion Island have a higher risk for death or severe morbidity compared with those born in mainland France.

**Electronic supplementary material:**

The online version of this article (10.1186/s12887-019-1700-7) contains supplementary material, which is available to authorized users.

## Background

In recent decades, advances in the management of severe prematurity have substantially reduced mortality in very preterm (VPT) infants, while also pushing the limit of viability to ever-lower gestational ages. However, neurodevelopmental sequelae are still a concern in children born VPT and their burden remains significantly high for the most immature infants [1–4].

Care practices and management of VPT infants differ between nations, regions within a same country, and hospital centres, explaining disparities in survival, especially at the extremes of viability [5]. Policies governing the organisation of health care and clinical guidelines are defined at the national level in France and are regionally implemented. However, between 2000 and 2008, infant mortality was twice as high in the French overseas regions (Départements d’Outre Mer, DOM: French Guyana, La Réunion, Guadeloupe and Martinique) than it was in mainland France (7.8 deaths per 1000 livebirths versus 3.8 per 1000) and perinatal pathologies accounted for two thirds of the excess mortality among DOM infants [6].

Although DOMs are shaped by large geographic and demographic discrepancies, they share a similar pattern of social deprivation that is worse than in mainland France. In agreement, pregnancy-related conditions in the DOMs differ from those observed in mainland France, as mothers are younger, more often isolated, and have a lower level of education [7].

In the French overseas department of La Réunion Island, VPT births (between 22 and 31 weeks + 6 days of gestation) are common (21 per 1000 livebirths in La Réunion Island versus 13 per 1000 in mainland France), neonatal mortality is more than two times higher than that observed in mainland France (4.9‰ versus 2.3‰), and VPT births account for more than half of this neonatal mortality [8].

With the aim of investigating the burden of VPT births, a population-based registry of VPT infants (Observatoire de la Grande Prématurité, OGP) was set up on La Réunion Island in 2008. In France, the latest large scale population-based cohort study of VPT infants is EPIPAGE-2 which enrolled participants in 2011 [9].

For the present study, our primary objective was to test the hypothesis that adverse neonatal outcomes of VPT infants were more likely observed in La Réunion Island than in mainland France. For this purpose, we compared the in-hospital neonatal mortality and severe neonatal morbidities in preterm infants born < 32 weeks of gestation (WG) from the La Réunion 2008–2013 registry-based OGP cohort with those included in the mainland 2011 EPIPAGE-2 cohort.

## Methods

### Population and setting

We enrolled in the study all VPT babies born between 24 WG and 31 WG + 6 days in two geographically defined cohorts and admitted alive in neonatal intensive care units (NICU). Infants born with severe birth defects were excluded.

The Reunionese OGP cohort enrolled VPT infants born in La Réunion Island (850,000 inhabitants and more than 14,000 births per year) between 2008 and 2013. Obstetrical and neonatal data were collected prospectively using standardised questionnaires by perinatal professionals in each of the seven maternity wards and the six neonatal care units of the island. This DOM includes two level-III neonatal care centres.

The EPIPAGE-2 cohort enrolled VPT infants born in year 2011 among 21 of the 22 mainland France’s regions (774,585 births). The duration of subject inclusion varied with gestational age (8-month recruitment period between 24 and 26 WG and 6-month recruitment period between 27 and 31 WG) [9]. EPIPAGE-2 infants native to other participating DOMs (Guadeloupe, Martinique, French Guiana) were excluded.

### Ethics

As required by French law and regulations, the OGP cohort was approved by the French national data protection authority (Comité National de l’Informatique et des Libertés, CNIL n°1250024). Families gave oral consent to participate in the cohort after they had received both oral and written information. Committee for the protection of people (CPP) agreement and written consent were not required for the OGP cohort, as monitoring of VPT infants is part of the standard care procedure and is not qualified as biomedical research according to national regulations.

The EPIPAGE-2 study was approved by the CNIL (CNIL n°911009) and by the appropriate ethics committees, i.e.*,* the advisory committee on the treatment of personal health data for research purposes (CCTIRS, approval granted November 18, 2010; reference number 10.626) and the CPP (approval granted March 18, 2011, reference CPP SC-2873). Families gave written informed consent to participate in the study after they had received both oral and written information [9].

### Outcome measures

The primary outcome was adverse neonatal outcomes, defined as a composite indicator reporting death during hospitalisation or survival at discharge complicated by at least one severe morbidity. Severe morbidity included bronchopulmonary dysplasia (BPD) and/or necrotising enterocolitis (NEC) and/or severe neurological injury. BPD (moderate to severe) was defined as oxygen dependency at 28 days of life plus treatment with oxygen and/or positive airway pressure at 36 weeks of postmenstrual age [10]. NEC was defined as Bell’s stage 2 or higher. Severe neurological injury was defined either as the presence of grade III or IV intraventricular haemorrhage (IVH), persistent hyperechogenicity on transfontanellar ultrasound (recorded only in EPIPAGE-2), or periventricular leukomalacia (PVL).

The secondary neonatal outcomes were each severe morbidity component of the primary composite outcome, as well as patent ductus arteriosus (PDA), treated either medically with non-steroidal anti-inflammatory drugs alone or with primary or secondary surgery, severe retinopathy of prematurity (ROP, stage ≥3), or late onset sepsis (LOS). In the aim of comparing the two cohorts, we standardised the definitions of morbidities as described in Additional file 1: Table S1.

### Explanatory variables

Obstetrical characteristics retrieved were maternal age, chronic or gestational arterial hypertension, pre-gestational or gestational maternal diabetes, premature prolonged rupture of membranes (occurring > 12 h before childbirth), antenatal steroid therapy (defined as any injection of corticosteroids before childbirth), multiple birth, and mode of delivery.

Neonatal data retrieved were gestational age (defined by a set of chronological, morphological and neurological criteria: last menstrual period, early ultrasound performed between 7 and 12 WG, morphological and neurological examination conducted at birth), birthweight, small for gestational age (SGA) status (defined as a birthweight ≤3rd percentile of the French AUDIPOG sentinel network growth charts), Apgar score < 7 at 5 min of life, outborn status, and surfactant therapy.

### Statistical analysis

We first analysed both cohorts separately to allow understanding of each cohort’s particularities. For an in-depth analysis of mortality and severe morbidity, we then compared the two cohorts, first as a whole, then by stratifying gestational age among three different groups: 24–26 WG, 27–28 WG, 29–31 WG. In this perspective, we ran different bivariable and multivariable analyses. For all these analyses, the EPIPAGE-2 cohort was taken as the reference cohort. Proportions in the EPIPAGE-2 cohort were weighted using appropriate sampling fractions to account for the disproportions between gestational age groups at inclusion in this study, as recommended for quota sampling.

Proportions were compared between the two cohorts using the chi-square tests, and means were compared using the Mann-Whitney test, or the Kruskal-Wallis test, as appropriate.

The risks associated with a very preterm birth occurring in La Réunion were estimated using weighted logistic regression models. Odds ratios (OR) and 95% confidence intervals (95% CI) were successively generated for the adverse neonatal outcomes (i.e., death, and/or BPD, and/or NEC and/or severe neurologic injury), then for each individual component of the primary outcome and secondary outcomes.

All the different models were adjusted on gestational age, gender, and a set of perinatal factors including maternal hypertension (chronic or pregnancy-induced) and diabetes mellitus (pre-gestational or gestational), antenatal steroid therapy, multiple birth, mode of delivery, outborn status, and 5-min Apgar score. All the interaction terms between the variables were tested. The adequacy of the models was tested using the Hosmer and Lemeshow goodness of fit test.

Statistical analysis was performed using SAS 9.4 (SAS Institute, Inc., Cary, NC). A *p* value less than 0.05 was considered statistically significant.

## Results

A total of 1272 VPT infants were enrolled in the Reunionese OGP cohort with a completeness > 95.0%, while in the mainland EPIPAGE-2 cohort, 3669 VPT infants were included and the participation was 96% [11].

### OGP cohort

First, we analysed the temporal trends of the primary composite outcome in relation to the obstetric and neonatal factors known to be associated with adverse neonatal outcome over three two-year time frames (2008–2009, 2010–2011 and 2012–2013) in the Additional file 2: Table S2. Overall, there was no significant change in the trends of mortality or severe morbidity over time. Subsequently, given the absence of significant changes in neonatal care during the study period, we considered that pooling the 6 years of the OGP cohort was not a source of bias for further analysis.

Second, we identified the variables associated with adverse neonatal outcomes in Reunionese VPT infants (Additional file 3: Table S3). In the OGP cohort, young mothers, absence of antenatal steroid therapy, singleton pregnancy, low gestational ages and birthweight, SGA status, low Apgar score < 7 and surfactant therapy were all associated with adverse neonatal outcomes.

### EPIPAGE-2 cohort

Similarly, we conducted such bivariable analysis of the primary composite outcome within the EPIPAGE-2 cohort (Additional file 4: Table S4). Factors associated with adverse neonatal outcomes were young mothers, maternal gestational diabetes, absence of antenatal steroids, vaginal delivery, low gestational age and birthweight, SGA status, male gender, low Apgar score < 7, and surfactant therapy.

### Obstetrical and neonatal characteristics of both cohorts

Reunionese mothers were younger than their mainland peers and were more frequently affected by hypertensive disorders and diabetes (Table 1). They were also more likely to receive antenatal steroids. Conversely, premature prolonged rupture of membranes, multiple birth, and caesarean delivery were more frequently observed in French mainland mothers.

Extremely immature infants (24–26 WG), low Apgar score < 7 and outborn status were less frequently reported in the OGP cohort than in the EPIPAGE-2 cohort and Reunionese infants were less likely to be treated with surfactant.

**Table 1:** Comparison of obstetrical and neonatal characteristics between the La Réunion Observatoire de la Grande Prématurité (OGP) 2008–2013 cohort and the EPIPAGE-2 2011 cohort

	OGP (*n* = 1272)		EPIPAGE 2 (*n* = 3669)		*P values*
	n	% or mean ± SE	n	% or mean ± SE	
Obstetrical characteristics					
Maternal age (years)	1115	28.2 ± 0.2	3669	29.7 ± 0.1	< 0.001
Maternal hypertension	290	29.3	849	24.6	0.003
Maternal diabetes	132	11.6	308	9.4	0.030
Premature prolonged rupture of membranes	204	22.3	1336	36.3	< 0.001
Antenatal steroid therapy	1022	89.7	2989	83.4	< 0.001
Multiple birth	299	23.5	1202	32.7	< 0.001
Caesarean delivery	709	60.8	2346	65.8	0.002
Neonatal characteristics					
Gestational age, weeks (WG)	1272	28.6 ± 0.06	3669	28.5 ± 0.03	0.201
24–26	222	17.5	791	21.6	0.008
27–28	307	24.1	842	22.9	
29–31	743	58.4	2036	55.5	
Birthweight (grams)	1271	1174.7 ± 9.6	3669	1194.2 ± 4.8	0.098
< 750	152	11.9	423	10.0	0.177
750–1000	290	22.8	875	22.0	
1000–1250	302	23.8	890	25.3	
≥ 1250	527	41.5	1481	42.7	
Small for gestational age^a^	180	14.2	467	12.9	0.253
Male sex	686	53.9	1933	52.7	0.446
Outborn status	76	6.0	533	14.5	< 0.001
Apgar score at 5 min < 7	69	7.4	646	18.0	< 0.001
Surfactant therapy	678	54.0	2427	65.1	< 0.001

^a^Birthweight <3rd percentile (French AUDIPOG growth charts)

### Adverse neonatal outcomes

In hospital mortality was higher in Reunionese than in mainland infants (15.4% versus 9.6%, *p* <  0.001). The primary composite outcome of death during hospitalisation or survival at discharge complicated of at least one severe morbidity was also more common in La Réunion (32.6% versus 26.6%, *p* <  0.001). Overall, severe morbidity was not different between the two databases, despite NEC was more likely among Reunionese VPT infants (Table 2). These findings were consistent across all gestational age strata for the primary composite outcome and its component fatal outcome. Conversely, LOS and medically-treated PDA were less likely in the OGP cohort than in the EPIPAGE-2 cohort (Table 3). In per strata analysis, BPD, surgically-treated PDA in the 29–31 WG subgroup and severe retinopathy across the more mature subgroups were more common in the OGP cohort.

**Table 2:** Comparison of neonatal outcomes between the La Réunion OGP 2008–2013 cohort and the EPIPAGE-2 2011 cohort: bivariable analysis

		OGP (*n* = 1272)		EPIPAGE 2 (*n* = 3669)		*P values*
		n	%	n	%	
Neonatal outcomes						
Death or severe morbidity^a^		415	32.6	1051	26.6	< 0.001
Death		196	15.4	396	9.6	< 0.001
Severe morbidity		219	21.4	655	19.6	0.214
Bronchopulmonary dysplasia		166	13.3	410	11.2	0.055
Necrotising enterocolitis		63	5.0	130	3.6	0.023
Severe neurological injury		110	9.1	401	10.3	0.207
Medical patent ductus arteriosus		129	10.3	726	19.4	< 0.001
Surgical patent ductus arteriosus		76	6.1	200	4.9	0.119
Retinopathy	Yes	29	2.3	36	0.8	< 0.001
	Missing data	926	72.8	983	27.2	
Late onset sepsis		351	28.2	1308	37.3	< 0.001

^a^severe morbidity = severe neurological injury, bronchopulmonary dysplasia or necrotising enterocolitis

**Table 3:** Comparison of neonatal outcomes between the La Réunion OGP 2008–2013 and the EPIPAGE-2 2011 cohorts by subgroups of gestational age: bivariable stratified analysis

Neonatal outcomes		Cohort	24–26 WG, % (n/N)	27–28 WG, % (n/N)	29–31 WG, % (n/N)
Death or severe morbidity^a^		OGP	74.3 (165/222)	44.0 (135/307)	15.5 (115/743)
		EPIPAGE 2	63.2 (500/791)**	35.3 (297/842)**	12.5 (254/2036)*
Death		OGP	45.9 (102/222)	19.2 (59/307)	4.7 (35/743)
		EPIPAGE 2	30.3 (240/791)***	12.1 (102/842)**	2.7 (54/2036)**
Severe morbidity^b^		OGP	55.3 (63/114)	32.5 (76/234)	11.8 (80/677)
		EPIPAGE 2	48.2 (260/539)	27.7 (195/704)	10.5 (200/1899)
Bronchopulmonary dysplasia		OGP	25.7 (56/218)	19.9 (60/301)	6.8 (50/732)
		EPIPAGE 2	32.0 (200/625)	16.8 (127/755)	4.2 (83/1972)**
Necrotising enterocolitis		OGP	6.9 (15/218)	7.3 (22/301)	3.6 (26/732)
		EPIPAGE 2	4.6 (35/761)	5.2 (42/812)	2.6 (53/2006)
Severe neurological injury		OGP	23.9 (51/213)	10.8 (31/287)	3.9 (28/711)
		EPIPAGE 2	26.2 (201/767)	11.4 (93/817)	5.4 (107/1993)
Medical patent ductus arteriosus		OGP	25.2 (55/218)	15.0 (45/301)	4.0 (29/733)
		EPIPAGE 2	40.7 (304/747)***	33.0 (267/809)***	7.9 (155/1969)***
Surgical patent ductus arteriosus		OGP	16.5 (36/218)	8.3 (25/301)	2.0 (15/732)
		EPIPAGE 2	18.6 (139/747)	5.3 (43/808)	0.9 (18/1956)*
Retinopathy	Yes	OGP	1.8 (4/222)	1.6 (5/307)	2.7 (20/743)
	Missing data		70.7 (157/222)	73.6 (226/307)	73.1 (543/743)
	Yes	EPIPAGE 2	3.7 (29/791)***	0.6 (5/842)***	0.1 (2/2036)***
	Missing data		20.3 (161/791)	31.1 (262/842)	27.5 (560/2036)
Late onset sepsis		OGP	56.0 (122/218)	42.2 (127/301)	14.0 (102/726)
		EPIPAGE 2	65.2 (473/726)*	49.4 (385/780)*	24.2 (450/1858)***

^a^Bronchopulmonary dysplasia, necrotising enterocolitis or severe neurological injury.^b^Defined as previously among survivors

Data are numbers and percentages (calculated on actual denominators after exclusion of missing data)

*P* values were calculated using a chi-squared test or a Fisher’s exact. *** *P* < 0.001; ** *P* < 0.01; and * *P* < 0.05

After adjusting on gestational age, gender and perinatal factors, the excess risk for adverse neonatal outcomes (i.e. composite outcome or death) in the OGP cohort was observed across all gestational age strata (Table 4). In per strata analysis, there were trends for the 27–28 WG and the 29-31WG Reunionese infants to be more affected by NEC or BPD, respectively. Conversely, Reunionese infants remained protected for medically treated PDA and LOS, irrespective of gestational age after adjustment on the abovementioned confounders.

**Table 4:** Comparison of neonatal outcomes between the La Réunion OGP 2008–2013 and the EPIPAGE-2 2011 cohorts by subgroups of gestational age: multivariable stratified analysis

Neonatal outcomes	24–26 WG	27–28 WG	29–31 WG
Death or severe morbidity^a^	2.3 (1.5–3.5)	1.5 (1.1–2.1)	1.3 (1.0–1.8)
Death	2.3 (1.6–3.4)	1.8 (1.2–2.8)	2.3 (1.4–3.8)
Severe morbidity^b^	1.7 (1.0–2.9)	1.4 (0.9–2.0)	1.1 (0.8–1.6)
Bronchopulmonary dysplasia	0.8 (0.5–1.2)	1.2 (0.8–1.8)	1.6 (1.0–2.5)
Necrotising enterocolitis^c^	1.4 (0.7–3.1)	1.8 (1.0–3.4)	1.4 (0.8–2.6)
Severe neurological injury	1.1 (0.7–1.7)	1.3 (0.8–2.2)	0.7 (0.4–1.2)
Medical patent ductus arteriosus	0.5 (0.3–0.7)	0.4 (0.3–0.6)	0.6 (0.4–1.0)
Surgical patent ductus arteriosus	1.0 (0.6–1.7)	1.9 (1.0–3.6)	2.0 (0.8–4.7)
Severe retinopathy	–	–	–
Late onset sepsis	0.7 (0.5–1.1)	0.7 (0.5–1.0)	0.5 (0.4–0.7)

Data are adjusted odds ratios with 95% confidence intervals in parenthesis as determined by logistic regression modelling

The reference cohort is EPIPAGE-2

All the different models were adjusted on gestational age, gender, and a set of perinatal factors including maternal hypertension (chronic or pregnancy-induced), maternal diabetes (pre-gestational or gestational), antenatal steroid therapy, multiple birth, mode of delivery, outborn status, and 5-min Apgar score

^a^Bronchopulmonary dysplasia, necrotising enterocolitis or severe neurological injury

^b^Defined as previously among survivors

^c^Same adjustment as previously, except for maternal diabetes due to lack of power

## Discussion

In this comparison of two population-based cohort studies, we demonstrate the more pejorative neonatal outcome of VPT infants born in La Réunion than in those native from mainland France. This finding was observed across the different subgroups of gestational age, after adjustment on most major confounding factors. In addition, mortality was higher in Reunionese infants, whichever the gestational age while the risks of bronchopulmonary dysplasia, severe retinopathy, and necrotizing enterocolitis were increased in specific subgroups of gestational age. Conversely, late onset sepsis and medically-treated patent ductus arteriosus were more common in mainland peers.

Numerous studies have compared mortality and the severe morbidity in VPT infants between different countries, regions, or networks. These adverse neonatal outcomes were highly variable [12–14]. Key components for comparing morbi-mortality in preterm babies cohorts include differences in maternal characteristics (age, socioeconomic factors, comorbidities, obstetrical history), infant characteristics (gestational age, birthweight, multiple birth, gender, ethnicity, outborn status, congenital anomaly, severity of illness on admission), prenatal and postnatal quality of care and adequacy to guidelines (prenatal care, obstetric management, perinatal health care organisation, physical and human characteristics of NICU), cultural and ethical considerations, definition and selection of outcomes, and population or referral characteristics [15–17].

The two cohorts compared in our study exhibited a relatively homogeneous population as evident from mean birthweight, gestational age, SGA status, and male gender which were not statistically different. Of note, the proportion of extremely immature infants in the 24–26 WG group was slightly higher in the EPIPAGE-2 cohort, given that its protocol favoured the recruitment of extremely immature infants [9]. Given the possibility of repartition bias, this methodological issue suggests caution in comparing the risk levels between the two databases taken overall. However, this was deemed not to be a significant limitation for comparing outcomes within each specific gestational age group.

By identifying pre-existing medical conditions, risk factors, and negative health behaviours through a range of medical and educational interventions, prenatal care can improve health outcomes for mothers and their infants. Interestingly, prenatal access to risk-appropriate neonatal care in La Réunion seemed adequate as suggested by higher proportions of inborn infants and antenatal steroid therapy. Importantly, these latter factors, recognized as life-saving interventions, are also protective against severe neonatal morbidities such as intraventricular haemorrhage in preterm infants [18–20]. Regionalisation of obstetrical care, especially prenatal transfer, may be facilitated by the small geographical size of the island and higher proportion of maternal disease in La Réunion. Though Reunionese women were younger, they were more prone to hypertensive disorders and diabetes mellitus. These findings are in agreement with the results of other studies in reproductive health, which show a poorer health status of ultramarine DOM populations [21, 22]. Indeed, maternal disease is often associated with excess mortality or with severe morbidities among preterm infants. Finally, the use of caesarean section as mode of delivery was higher in the EPIPAGE-2 cohort, despite lesser maternal morbidity. Caesarean section has been shown to improve the survival for extremely preterm infants as well as for VPT infants aged between 26 and 30 WG suffering intrauterine growth restriction [23]. A higher rate of caesarean section may also indicate a more proactive antenatal management of very high-risk pregnancies. In our study, we did not find any association between caesarean section and adverse neonatal outcomes in the OGP cohort, while it was slightly protective in the EPIPAGE-2 cohort. However, given the excess adverse neonatal outcomes in the 24–26 WG subgroup in the OGP cohort, more proactive perinatal management should be considered for improving Reunionese infant’s outcomes [24].

The burden of BPD was higher in the OGP cohort beyond the age of 28 WG, but it was not different in the 24–26 and 27–28 WG subgroups, perhaps as a consequence of higher antenatal steroid therapy coverage in these groups at-risk for chronic lung disease. These results may also illustrate the higher fatality rate in the most immature VPT infants and a selective survival bias, mortality and BPD risks being competitive and better survival observed in low-risk infants. In support of our previous results, these new findings suggest that better respiratory and nutritional strategies starting from the delivery room and continued in the neonatal care unit are locally warranted [25, 26].

The risk of NEC was increased in the OGP cohort, but only in the 27–28 WG stratum. This finding may uncover the combination of several risk factors of NEC in Reunionese infants, among which lower Apgar score, hypoxia-ischemia and compromised respiratory function limiting nutrient and oxygen uptake to the gut [27]. Human milk feeding is associated with a lower risk of NEC and is being increasing recognized as protective against BPD [28]. An as yet unexamined hypothesis is the possibility of lesser than expected rate of breastfeeding, or use of human milk, in Reunionese VPT infants. In support to this idea, only 30% of Reunionese mothers breastfed their VPT infants at hospital discharge (personal unpublished data). Moreover, there is no human milk bank available on the island where NICUs have to import pasteurized banked donor milk from mainland France. The lower prevalences of BPD and NEC, together with a higher expected rate of breastfeeding in the EPIPAGE-2 cohort, should impel the promotion of breastfeeding for Reunionese mothers [28].

The incidence of medically-treated PDA was lower in the OGP cohort. PDA is associated with an increased mortality in VPT infants, and its role in the development of BPD remains controversial. Even though optimal management of PDA remains a matter of debate, a recent study within the EPIPAGE-2 cohort revealed that early screening echocardiography was predictive of more frequent treatment and lower in-hospital mortality. Conversely, there was no association between severe BPD and early screening echocardiography [29].

We found difficult to compare the burden of severe ROP between the two cohorts, as more than three quarters of VPT infants did not undergo fundus examination in the OGP cohort. Indeed, the statistical association found in the bivariable analysis between severe ROP and the absence of adverse neonatal outcomes in the OGP cohort was more likely the result of another survival selective bias.

Late onset sepsis was less common among Reunionese than among mainland VPT infants. This result was observed with a stringent definition of LOS prevailing for the bacteriological evidence of infection or at least a five-day antibiotic treatment. A less restrictive definition including possible infections, for which bacterium was not proved or antibiotic treatment was discontinued, found Reunionese infants more likely infected. Given the inconsistence of the findings, it would be hazardous to conclude that the higher burden of LOS in the EPIPAGE-2 cohort may be related to lesser maturity.

Our study has some strengths and limitations. First, we studied two nearly contemporaneous populations with a high completeness rate and large sample sizes. Second, we chose a composite outcome as primary outcome measure to limit the survival bias as all severe morbidity outcomes compete with mortality in this population, and because improved survival of extremely immature preterm infants may increase the burden of short and long-morbidities [1–4]. Third, stratified and multivariate analyses allowed testing the consistency of the associations across all gestational age strata, while adjusting for major confounders to provide accurate risk estimates. Finally, to the best of our knowledge, this is the first study detailing the characteristics and short-term outcomes of VPT infants from a French remote overseas territory and allowing two different ethnic and genetic backgrounds benefiting from the same health care system to be compared.

Limitations of our study consisted essentially in an information bias. First, we used a restrictive set of individual variables, especially maternal socio-economic characteristics missing in the OGP cohort, which prevented thorough explanation of population differences. Indeed, health inequalities are a major contributor of prematurity in French overseas departments, but it remains to be shown whether social deprivation also contribute to VPT outcomes [30]. Second the absence of a neonatal illness severity score in both cohorts hampered assessment of the severity of initial neonatal disease and its further involvement in discharge outcomes. Third, we did not retrieve information regarding prenatal and postnatal quality of care such as potential work overloads, inadequate staffing, and neonatal policies in cardiorespiratory, nutritional, and ethical management which may also influence our primary composite outcome [31]. Indeed, given a lack of information, we were unable to further investigate the relationships between severe neurologic injury and death under the circumstances in which end-of-life treatment may be ethically and legally limited.

## Conclusion

In conclusion, adverse neonatal outcomes were more common in Reunionese than in mainland France VPT infants, regardless the gestational age group. Furthermore, we identified some discrepancies in various individual outcomes. Recognizing outcome variations in VPT infants across different settings may provide valuable information for identifying areas of improvement within each country. Better management of maternal diseases, more proactive antenatal care in extremely premature infants, less aggressive respiratory support, and enhanced breastfeeding policy, could improve the outcome of VPT birth in La Réunion and other DOM populations. Such a comparative study using standardised definitions of adverse neonatal outcomes should therefore be extended to other remote ultramarine areas to guide public health interventions aimed at improving perinatal health indicators, known to be pejorative in these regions.

## Additional files

Additional file 1: **Table S1.** Consensual definitions of neonatal outcomes between the Observatoire de La Grande Prématurité, La Réunion (OGP) cohort and the EPIPAGE 2 mainland France cohort. (DOCX 17 kb)

Additional file 2: **Table S2.** Trends for obstetrical and neonatal characteristics and neonatal outcomes in the OGP 2008–2013 cohort. (DOCX 29 kb)

Additional file 3: **Table S3.** Adverse neonatal outcomes according to other severe morbidities, obstetrical and neonatal characteristics in the OGP 2008–2013 cohort. (DOCX 25 kb)

Additional file 4: **Table S4.** Adverse neonatal outcomes according to other severe morbidities, obstetrical and neonatal characteristics in the EPIPAGE-2 2011 cohort. (DOCX 27 kb)

## Data Availability

The EPIPAGE 2 studies are subject to a data sharing policy that may be downloaded from http://epipage2.inserm.fr/index.php/en/. For the OGP cohort, the datasets used and/or analysed during the current study are available from the corresponding author on reasonable request.

## Abbreviations

BPD: Bronchopulmonary dysplasia
CNIL: Comité National de l’Informatique et des Libertés
CPP: Commitee for the protection of people
DOM: Départements d’Outre Mer
IVH: Intraventricular haemorrhage
LOS: Late onset sepsis
NEC: Necrotising enterocolitis
NICU: Neonatal intensive care units
OGP: Observatoire de Grande Prématurité
PDA: Patent ductus arteriosus
PVL: Periventricular leukomalacia
ROP: Retinopathy of prematurity
SGA: Small for gestational age
VPT: Very preterm infants
WG: Weeks of gestation
