# Supplementary material for: Mortality and severe morbidity of very preterm infants: comparison of two French cohort studies
Source: BMC Pediatr. 2019 Oct 17;19:360. doi: 10.1186/s12887-019-1700-7 (PMC6796444; doi:10.1186/s12887-019-1700-7)
Supplement: Supplementary file 1 — Table S1. Consensual definitions of neonatal outcomes between the Observatoire de La Grande Prématurité, La Réunion (OGP) cohort and the EPIPAGE 2 mainland France cohort. (DOCX 17 kb) [file 12887_2019_1700_MOESM1_ESM.docx]

**Table S1. Consensual definitions of neonatal outcomes between the Observatoire de La Grande Prématurité, La Réunion (OGP) cohort and the EPIPAGE 2 mainland France cohort.**

| **Neonatal outcomes** | **OGP** | **EPIPAGE 2** | **Consensus** |
| --- | --- | --- | --- |
| Mortality | Death before last discharge from neonatal hospitalisation | Death before last discharge from neonatal hospitalisation | Death before last discharge from neonatal hospitalisation |
| Bronchopulmonary dysplasia | Oxygen or positive airway pressure at 36 WG corrected age | Oxygen or positive airway pressure at 36 WG corrected age | Oxygen or positive airway pressure at 36 WG corrected age |
| Necrotising enterocolitis | According to Bell’s criteria stage 2 or higher | According to Bell’s criteria stage 2 or higher | According to Bell’s criteria stage 2 or higher |
| Severe neurological injury | IVH grade III-IV or PVL | IVH grade III-IV or IPH | IVH grade III-IV or PVL or IPH |
| Medical patent ductus arteriosus | Ductus arteriosus treated with ibuprofen or indomethacin without surgery | Ductus arteriosus treated with ibuprofen or indomethacin without surgery | Ductus arteriosus with medical treatment alone |
| Surgical patent ductus arteriosus | Ductus arteriosus treated with surgery irrespective of medical treatment | Ductus arteriosus treated with surgery irrespective of medical treatment | Ductus arteriosus treated with surgery irrespective of medical treatment |
| Severe retinopathy | All-stage retinopathy | Stage 3-4 | Stage 3-4 or laser-treated |
| Late onset sepsis | Confirmed or possible nosocomial infection if treated ≥ 5 days | Secondary infection during neonatal care | Secondary infection during neonatal care confirmed and/or treated ≥ 5 days |

WG: weeks of gestation; IVH: intraventricular haemorrhage; PVL: periventricular leukomalacia; IPH: persistent intraparenchymal hyperechogenicity
