# Supplementary material for: Mortality and severe morbidity of very preterm infants: comparison of two French cohort studies
Source: BMC Pediatr. 2019 Oct 17;19:360. doi: 10.1186/s12887-019-1700-7 (PMC6796444; doi:10.1186/s12887-019-1700-7)
Supplement: Supplementary file 2 — Table S2. Trends for obstetrical and neonatal characteristics and neonatal outcomes in the OGP 2008–2013 cohort. (DOCX 29 kb) [file 12887_2019_1700_MOESM2_ESM.docx]

**Table S2: Trends for obstetrical and neonatal characteristics and neonatal outcomes in the OGP 2008-2013 cohort.**

|  | **OGP**  **(n = 1,272)** | | **2008-2009**  **(n = 432)** | | **2010-2011**  **(n = 391)** | | **2012-2013**  **(n = 449)** | |  |
| --- | --- | --- | --- | --- | --- | --- | --- | --- | --- |
|  | **n** | **% or**  **mean ± SE** | **n** | **% or**  **mean ± SE** | **n** | **% or**  **mean ± SE** | **n** | **% or**  **mean ± SE** | *P* values |
| **Neonatal outcome** |  |  |  |  |  |  |  |  |  |
| Death or severe morbidity ^a^ | 415 | 32.6 | 127 | 29.4 | 124 | 31.7 | 164 | 36.5 | 0.071 |
| Death | 196 | 15.4 | 57 | 13.2 | 65 | 16.6 | 74 | 16.5 | 0.292 |
| Severe morbidity | 219 | 21.4 | 70 | 20.3 | 59 | 18.8 | 90 | 24.5 | 0.166 |
| Severe neurological injury | 110 | 9.1 | 30 | 7.5 | 32 | 8.6 | 48 | 10.9 | 0.219 |
| Bronchopulmonary dysplasia | 166 | 13.3 | 54 | 12.7 | 44 | 11.5 | 68 | 15.4 | 0.231 |
| Necrotising enterocolitis | 63 | 5.0 | 18 | 4.2 | 15 | 3.9 | 30 | 6.8 | 0.106 |
| Retinopathy Yes | 29 | 2.3 | 11 | 2.6 | 9 | 2.3 | 9 | 2.0 | 0.987 |
| MD | 926 | 72.8 | 312 | 72.2 | 285 | 72.9 | 329 | 73.3 |  |
| Late neonatal sepsis | 351 | 28.2 | 129 | 30.4 | 97 | 25.3 | 125 | 28.5 | 0.269 |
| Medical patent ductus arteriosus | 129 | 10.3 | 36 | 8.5 | 36 | 9.4 | 57 | 12.9 | 0.077 |
| Surgical patent ductus arteriosus | 76 | 6.1 | 26 | 6.1 | 30 | 7.8 | 20 | 4.5 | 0.148 |
| **Obstetrical characteristics** |  |  |  |  |  |  |  |  |  |
| Maternal age, years | 1,115 | 28.2 ± 0.2 | 375 | 28.2 ± 0.4 | 366 | 28.6 ± 0.4 | 374 | 27.6 ± 0.4 | 0.114^b^ |
| Maternal hypertension | 290 | 29.3 | 86 | 26.7 | 100 | 31.0 | 104 | 30.1 | 0.456 |
| Gestational diabetes | 103 | 9.1 | 28 | 7.1 | 43 | 11.7 | 32 | 8.5 | 0.081 |
| Pregestational diabetes | 30 | 2.6 | 9 | 2.3 | 11 | 3.0 | 10 | 2.6 | 0.834 |
| Maternal diabetes | 132 | 11.6 | 37 | 9.4 | 54 | 14.7 | 41 | 10.9 | 0.066 |
| Premature prolonged rupture of  membranes | 204 | 22.3 | 78 | 24.8 | 65 | 23.0 | 61 | 19.3 | 0.245 |
| Antenatal steroid therapy | 1,022 | 89.7 | 357 | 90.6 | 332 | 90.2 | 333 | 88.3 | 0.541 |
| Multiple birth | 299 | 23.5 | 111 | 25.7 | 91 | 23.3 | 97 | 21.6 | 0.356 |
| Caesarean delivery | 709 | 60.8 | 232 | 58.9 | 225 | 61.1 | 252 | 62.4 | 0.593 |
| **Neonatal characteristics** |  |  |  |  |  |  |  |  |  |
| Gestational age, weeks | 1,272 | 28.6 ± 0.06 | 432 | 28.6 ± 0.10 | 391 | 28.6 ± 0.10 | 449 | 28.8 ± 0.09 | 0.121 |
| 24 - 26 | 222 | 17.5 | 75 | 17.4 | 70 | 17.9 | 77 | 17.1 | 0.461 |
| 27 - 28 | 307 | 24.1 | 114 | 26.4 | 97 | 24.8 | 96 | 21.4 |  |
| 29 - 31 | 743 | 58.4 | 243 | 56.2 | 224 | 57.3 | 276 | 61.5 |  |
| Birthweight, grams | 1,271 | 1,174.7 ± 9.6 | 432 | 1,150.3 ± 7.5 | 391 | 1,179.0 ± 18.4 | 448 | 1,194.6 ± 17.2 | 0.149 |
| <750 | 152 | 11.9 | 56 | 13.0 | 41 | 10.5 | 55 | 12.3 | 0.619 |
| 750-1000 | 290 | 22.8 | 105 | 24.3 | 94 | 24.0 | 91 | 20.3 |  |
| 1000-1250 | 302 | 23.8 | 104 | 24.1 | 90 | 23.0 | 108 | 24.1 |  |
| ≥1250 | 527 | 41.5 | 167 | 38.6 | 166 | 42.5 | 194 | 43.3 |  |
| SGA (birthweight <3^rd^ percentile) | 180 | 14.2 | 68 | 15.7 | 45 | 11.5 | 67 | 14.9 | 0.186 |
| Male gender | 686 | 53.9 | 244 | 56.5 | 202 | 51.7 | 240 | 53.5 | 0.371 |
| Outborn status | 76 | 6.0 | 34 | 7.9 | 20 | 5.1 | 22 | 4.9 | 0.122 |
| Apgar score at 5 min. < 7 | 69 | 7.4 | 15 | 4.8 | 26 | 8.2 | 28 | 9.2 | 0.091 |
| Surfactant therapy | 678 | 54.0 | 234 | 54.5 | 208 | 54.0 | 236 | 53.4 | 0.943 |

^a^ severe morbidity = severe neurological injury, bronchopulmonary dysplasia or necrotising enterocolitis.

^b^ Fisher’s exact test for qualitative variables or non-parametric Kruskal-Wallis test for quantitative variables.

MD: missing data; min.: minute; SGA: small for gestational age
