# Supplementary material for: Mortality and severe morbidity of very preterm infants: comparison of two French cohort studies
Source: BMC Pediatr. 2019 Oct 17;19:360. doi: 10.1186/s12887-019-1700-7 (PMC6796444; doi:10.1186/s12887-019-1700-7)
Supplement: Supplementary file 3 — Table S3. Adverse neonatal outcomes according to other severe morbidities, obstetrical and neonatal characteristics in the OGP 2008–2013 cohort. (DOCX 25 kb) [file 12887_2019_1700_MOESM3_ESM.docx]

**Table S3. Adverse neonatal outcomes according to other severe morbidities, obstetrical and neonatal characteristics in the OGP 2008-2013 cohort.**

|  | **OGP**  **(n=1,272)** | | **Survival without severe morbidity (n=857)** | | **Death or severe**  **morbidity (n=415)** | | ***P values*** |
| --- | --- | --- | --- | --- | --- | --- | --- |
|  | **n** | **% or**  **mean ± SE** | **n** | **% or**  **mean ± SE** | **n** | **% or**  **mean ± SE** |  |
| **Neonatal outcomes** |  |  |  |  |  |  |  |
| Medical patent ductus arteriosus | 129 | 10.3 | 61 | 7.3 | 68 | 16.4 | < 0.001 |
| Surgical patent ductus arteriosus | 76 | 6.1 | 22 | 2.6 | 54 | 13.0 | < 0.001 |
| Retinopathy Yes | 29 | 2.3 | 26 | 3.0 | 3 | 0.7 | 0.019 |
| Missing data | 926 | 72.8 | 627 | 73.2 | 299 | 72.1 |  |
| Late onset sepsis | 351 | 28.2 | 151 | 18.2 | 200 | 48.3 | < 0.001 |
| **Obstetrical characteristics** |  |  |  |  |  |  |  |
| Maternal age (years) | 1,115 | 28.2 ± 0.2 | 747 | 28.5 ± 0.2 | 368 | 27.5 ± 0.4 | 0.016 |
| Maternal hypertension | 290 | 29.3 | 203 | 30.9 | 87 | 26.1 | 0.113 |
| Maternal diabetes | 132 | 11.6 | 93 | 12.2 | 39 | 10.5 | 0.392 |
| Premature prolonged rupture of  membranes | 204 | 22.3 | 144 | 23.6 | 60 | 19.7 | 0.186 |
| Antenatal steroid therapy | 1,022 | 89.7 | 700 | 91.1 | 322 | 86.8 | 0.023 |
| Multiple birth | 299 | 23.5 | 216 | 25.2 | 83 | 20.0 | 0.040 |
| Caesarean delivery | 709 | 60.8 | 482 | 61.5 | 227 | 59.4 | 0.500 |
| **Neonatal characteristics** |  |  |  |  |  |  |  |
| Gestational age, weeks (WG) | 1,272 | 28.6 ± 0.06 | 857 | 29.3 ± 0.06 | 415 | 27.3 ± 0.09 | < 0.001 |
| 24 - 26 | 222 | 17.5 | 57 | 6.6 | 165 | 39.8 | < 0.001 |
| 27 - 28 | 307 | 24.1 | 172 | 20.1 | 135 | 32.5 |  |
| 29 - 31 | 743 | 58.4 | 628 | 73.3 | 115 | 27.7 |  |
| Birthweight (grams) | 1,271 | 1,174.7 ± 9.7 | 856 | 1,274.4 ± 11.5 | 415 | 969.1 ± 16.5 | < 0.001 |
| <750 | 152 | 11.9 | 34 | 4.0 | 118 | 28.4 | < 0.001 |
| 750-1000 | 290 | 22.8 | 149 | 17.4 | 141 | 34.0 |  |
| 1000-1250 | 302 | 23.8 | 225 | 26.3 | 77 | 18.6 |  |
| >1250 | 527 | 41.5 | 448 | 52.3 | 79 | 19.0 |  |
| Small for gestational age ^a^ | 180 | 14.2 | 86 | 10.0 | 94 | 22.7 | < 0.001 |
| Male gender | 686 | 53.9 | 449 | 52.4 | 237 | 57.1 | 0.114 |
| Outborn status | 76 | 6.0 | 54 | 6.3 | 22 | 5.3 | 0.481 |
| Apgar score at 5 min < 7 | 69 | 7.4 | 36 | 5.7 | 33 | 10.8 | 0.005 |
| Surfactant therapy | 678 | 54.0 | 357 | 42.4 | 321 | 77.3 | < 0.001 |

Data are numbers and column percentages (calculated on actual denominators after exclusion of missing data)

^a^ Birthweight < 3^rd^ percentile (French AUDIPOG growth charts).

*P* values were calculated using a chi-squared test for percentages, or using a Mann-Whitney test for means.
