# Supplementary material for: Mortality and severe morbidity of very preterm infants: comparison of two French cohort studies
Source: BMC Pediatr. 2019 Oct 17;19:360. doi: 10.1186/s12887-019-1700-7 (PMC6796444; doi:10.1186/s12887-019-1700-7)
Supplement: Supplementary file 4 — Table S4. Adverse neonatal outcomes according to other severe morbidities, obstetrical and neonatal characteristics in the EPIPAGE-2 2011 cohort. (DOCX 27 kb) [file 12887_2019_1700_MOESM4_ESM.docx]

**Table S4. Adverse neonatal outcomes according to other severe morbidities, obstetrical and neonatal characteristics in the EPIPAGE-2 2011 cohort.**

|  | **EPIPAGE2**  **(n=3,669)** | | | **Survival without**  **severe morbidity**  **(n=2,618)** | | **Death or severe**  **morbidity**  **(n=1,051)** | | ***P values*** |
| --- | --- | --- | --- | --- | --- | --- | --- | --- |
|  | **n** | | **% or**  **mean ± SD** | **n** | **% or**  **mean ± SD** | **n** | **% or**  **mean ± SD** |  |
| **Neonatal outcomes** | |  |  |  |  |  |  |  |
| Medical patent ductus arteriosus | | 726 | 19.4 | 403 | 15.1 | 323 | 31.8 | < 0.001 |
| Surgical patent ductus arteriosus | | 200 | 4.9 | 67 | 2.3 | 133 | 12.4 | < 0.001 |
| Retinopathy Yes | | 36 | 0.8 | 9 | 0.3 | 27 | 2.3 | < 0.001 |
| Missing data | | 983 | 27.2 | 772 | 29.5 | 211 | 20.6 |  |
| Late onset sepsis | | 1,308 | 37.3 | 766 | 30.5 | 542 | 56.7 | < 0.001 |
| **Obstetrical characteristics** | |  |  |  |  |  |  |  |
| Maternal age (years) | | 3,669 | 29.7 ± 0.1 | 2,618 | 29.9 ± 0.1 | 1,051 | 29.2 ± 0.2 | 0.001 |
| Maternal hypertension | | 849 | 24.6 | 620 | 24.9 | 229 | 23.9 | 0.568 |
| Maternal diabetes | | 308 | 9.4 | 239 | 10.1 | 69 | 7.4 | 0.018 |
| Premature prolonged rupture of membranes | | 1,336 | 36.3 | 961 | 36.7 | 375 | 35.1 | 0.358 |
| Antenatal steroid therapy | | 2,989 | 83.4 | 2,194 | 85.4 | 795 | 77.8 | < 0.001 |
| Multiple birth | | 1,202 | 32.7 | 858 | 32.9 | 344 | 32.1 | 0.636 |
| Caesarean delivery | | 2,346 | 65.8 | 1,723 | 67.1 | 623 | 62.2 | 0.005 |
| **Neonatal characteristics** | |  |  |  |  |  |  |  |
| Gestational age, weeks (WG) | | 3,669 | 28.5 ± 0.03 | 2,618 | 29.1 ± 0.04 | 1,051 | 27.0 ± 0.06 | < 0.001 |
| 24 - 26 | | 791 | 21.6 | 291 | 11.1 | 500 | 47.6 | < 0.001 |
| 27 - 28 | | 842 | 22.9 | 545 | 20.8 | 297 | 28.2 |  |
| 29 - 31 | | 2,036 | 55.5 | 1,782 | 68.1 | 254 | 24.2 |  |
| Birthweight, grams | | 3,669 | 1,194.2 ± 4.8 | 2,618 | 1,274.9 ± 5.8 | 1,051 | 971.8 ± 9.1 | < 0.001 |
| <750 | | 423 | 10.0 | 128 | 4.3 | 295 | 25.8 | < 0.001 |
| 750-1000 | | 875 | 22.0 | 496 | 17.6 | 379 | 34.2 |  |
| 1000-1250 | | 890 | 25.3 | 680 | 26.4 | 210 | 22.0 |  |
| >1250 | | 1,481 | 42.7 | 1,314 | 51.7 | 167 | 18.0 |  |
| Small for gestational age ^a^ | | 467 | 12.9 | 274 | 10.6 | 193 | 19.1 | < 0.001 |
| Male gender | | 1933 | 52.7 | 1,344 | 51.5 | 589 | 56.1 | 0.011 |
| Outborn status | | 533 | 14.5 | 363 | 14.0 | 170 | 16.1 | 0.100 |
| Apgar score at 5 min. < 7 | | 646 | 18.0 | 362 | 14.2 | 284 | 28.7 | < 0.001 |
| Surfactant therapy | | 2,427 | 65.1 | 1,494 | 56.6 | 933 | 88.7 | < 0.001 |

Data are numbers and column percentages (calculated on actual denominators after exclusion of missing data).

^a^ Birthweight < 3^rd^ percentile (French AUDIPOG growth charts).

*P* values were calculated using a chi-squared test for percentages, or using a Mann-Whitney test for means.
